# Reliable and early diagnosis of bacterial blight in pomegranate caused by *Xanthomonas axonopodis* pv. *punicae* using sensitive PCR techniques

**DOI:** 10.1038/s41598-019-46588-9

**Published:** 2019-07-12

**Authors:** Pushpa Doddaraju, Pavan Kumar, Raghavendra Gunnaiah, Abhishek A. Gowda, Veeresh Lokesh, Parvati Pujer, Girigowda Manjunatha

**Affiliations:** 1 Bio-control Lab, Directorate of Research, University of Horticultural Sciences, Bagalkot, Karnataka India; 2 Department of Biotechnology and Crop Improvement, University of Horticultural Sciences, Bagalkot, Karnataka India

**Keywords:** Plant molecular biology, Biotic, High-throughput screening

## Abstract

Bacterial blight caused by *Xanthomonas axonopodis* pv. *punicae* is a major disease of pomegranate. Bacterial blight drastically reduces the yield and quality of fruits, which are critical for pomegranate production. Precise and early diagnosis of bacterial blight is crucial for active surveillance and effective management of the disease. Symptoms based disease diagnostic methods are labor-intensive, time-consuming and may not detect disease on asymptomatic plants. DNA-based disease diagnostics using polymerase chain reaction (PCR) are reliable, precise, accurate and quick. PCR coupled with agarose gel electrophoresis (PCR-AGE), PCR coupled with capillary electrophoresis (PCR-CE) and real-time PCR (qPCR) were applied for the early and accurate diagnosis of bacterial blight in pomegranate. PCR-CE and qPCR were capable of diagnosing bacterial blight 6 to 10 days before symptom appearance, with detection limits of 100 fg and 10 fg of bacterial DNA respectively. However, conventional PCR-AGE detected pathogen at the onset of disease symptoms with a detection limit of 10 pg of bacterial DNA. qPCR detected bacterial blight in orchards that did not show any disease symptoms. Our data demonstrate that qPCR is more sensitive than other PCR methods along with being reliable for early diagnosis.

## Introduction

Pomegranate is an important fruit crop of subtropical and tropical regions of the world and is promoted as a functional food and nutraceutical source with health promoting benefits^1^. Additionally, the long shelf life of pomegranate encourages huge demand in domestic and international markets. India is the largest producer of pomegranate in the world with an annual production of 2,442 thousand tonnes grown in 209 thousand hectares^2^. The annual pomegranate fruit export is approximately 35,000 tonnes^3^. Bacterial blight caused by *Xanthomonas axonopodis* pv. *punicae* (*Xap*) is a major constraint of pomegranate cultivation and production^4^. Bacterial blight mostly affects above ground parts of pomegranate such as leaves, twigs, and fruits^5^. The initial water-soaked lesions appear only after 6 to 7 days of infection under favourable field conditions and develop into late necrotic blighting^6^. Fruits exhibit isolated water-soaked lesions followed by necrosis with small cracks, leading to splitting of the entire fruit. Severe disease outbreaks can cause 60 to 80% yield losses^5^. Bacterial blight is gaining international attention through its recent spread to other major growing areas of the world such as Pakistan^7^, South Africa^8^, and Turkey^9^.

*Xap* is a gram negative, rod shaped bacterium that measures 0.4 to 0.75 × 1.0 to 3.0 μm with single polar flagellum^10^. *Xap* is cultured *in vitro* on different synthetic media; peptone yeast extract dextrose media, nutrient glucose agar and Luria-Bertani media. *Xap* produces smooth, circular, light yellow, glistening mucoid, butyrous and convex colonies with entire margins. *Xap* genome (4.94 Mb) is >99% identical to *Xanthomonas axonopodis* pv. *citri*, encodes 4,385 protein coding genes, 50 tRNA and 3 rRNA genes^11^. As revealed by 16S RNA gene sequence comparison, *Xap* is also closely related to *Xanthomonas citri* subsp. *malvacearum* and *X*. *axonopodis* pv. *manihotis*. *Xap* survives in infected plant parts and debris in the soil up to one year and spreads through rain splashes, irrigation water, pruning tools, insect vectors and human beings^10^. The bacterium enters and infects different parts of plant through natural openings like stomata, lenticels, hydathodes or wounds. Bacterial blight symptoms on pomegranate appear after 3 to 4 days of challenge infection under favourable conditions (30 °C, 60–70% relative humidity) and continue to develop up to 30 days depending upon incubation factors^5^. *Xap* also survives in latent form in the healthy plant parts obtained from diseased trees and produce disease symptoms even after seven months of incubation^10^. Rainy season (July to September) with temperature of 25 °C to 35 °C and relative humidity of >30% is highly conducive for *Xap* infection in pomegranate. *Xap* secretes effector proteins *XopN* and *XopL* by the type III secretion system (T3SS) for pathogenesis and to suppress host immune responses^12,13^.

Bacterial blight of pomegranate is managed by cultural, chemical and biological methods. Measures to reduce disease pressure include: planting disease free planting material; avoiding flowering and fruiting during excessive rainy periods; complete defoliation after harvest of the crop followed by 3–4 months of rest period and maintaining orchard sanitation standards^14^. Streptocycline (streptomycin sulphate, 500 ppm) in combination with copper oxychloride (0.2%) followed by Bronopol (2-bromo-2-nitropropane-1,3-diol, 500 ppm) and copper oxychloride (0.2%) were found to be effective in the management of bacterial blight of pomegranate^15,16^. Alternative chemicals and biologicals, such as spraying plant growth regulators ethylene (200 ppm)^17^, nano copper at 2 ppm^18^, bacterial antagonistic such as *Pseudomonas fluorescens* and *Bacillus subtilis*^19^, *Streptomyces violaceusnige*^20^ and plant extracts from pongamia, neem, coleus and periwinkle have been shown to effectively retard the growth of *Xap* under *in vitro* conditions^21^. Integrated disease management with cultural practices, orchard sanitation, timely application of pesticides along with biologicals and botanicals are effective in reducing bacterial blight severity in pomegranate^16^.

Precise and early diagnosis of bacterial blight is crucial for effective management of the disease. Diagnosis of bacterial blight in pomegranate is based on phenotypic disease symptoms^6^. In the absence of favourable environment, the symptoms are often masked as the pathogen survives in the latent form and the disease is unnoticed in the field at early days of infection. Early detection of the pathogen during the latent phase in fields and nurseries would help in prevention, efficient management, and restricting further spread of the disease.

Many advanced molecular techniques have been used for detection and quantification of bacteria^22^. The DNA-based polymerase chain reaction (PCR) has been used for the detection of bacterial blight in pomegranate^23^. PCR coupled with capillary electrophoresis (PCR-CE)^24^ and real-time quantitative PCR (qPCR) have been shown to be rapid and more sensitive methods for detection of pathogens in host plants at early phases of infection^25^. In this study, PCR and qPCR based disease diagnostics methods were compared for accurate and early diagnosis of bacterial blight in pomegranate during latent infection stages.

## Results

### Selection of genes for *Xap* detection and specificity of the assay

**Figure 1:**
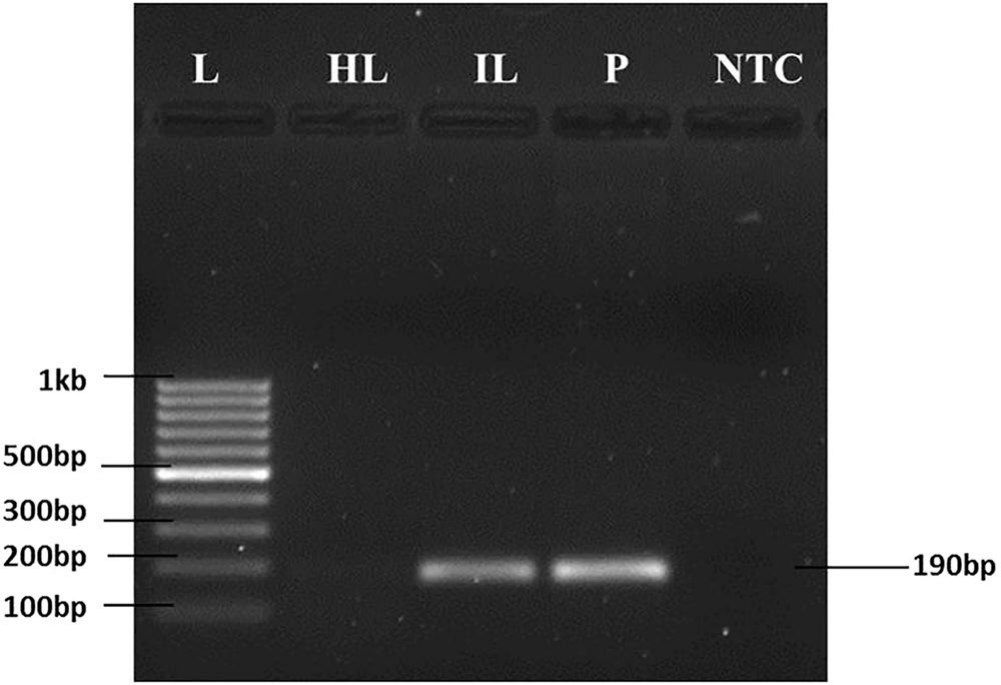
Assessment of primer specificity in detecting *Xanthomonas axonopodis* pv. *punicae* (*Xap*). Specific amplification of effector gene *XopQ* in Healthy leaves, *Xap* infected leaves and pure culture of *Xap*. L-100 bp ladder, HL-healthy leaf, IL-infected leaf, P- cultured *Xap*, and NTC- no template control.

**Figure 2:**
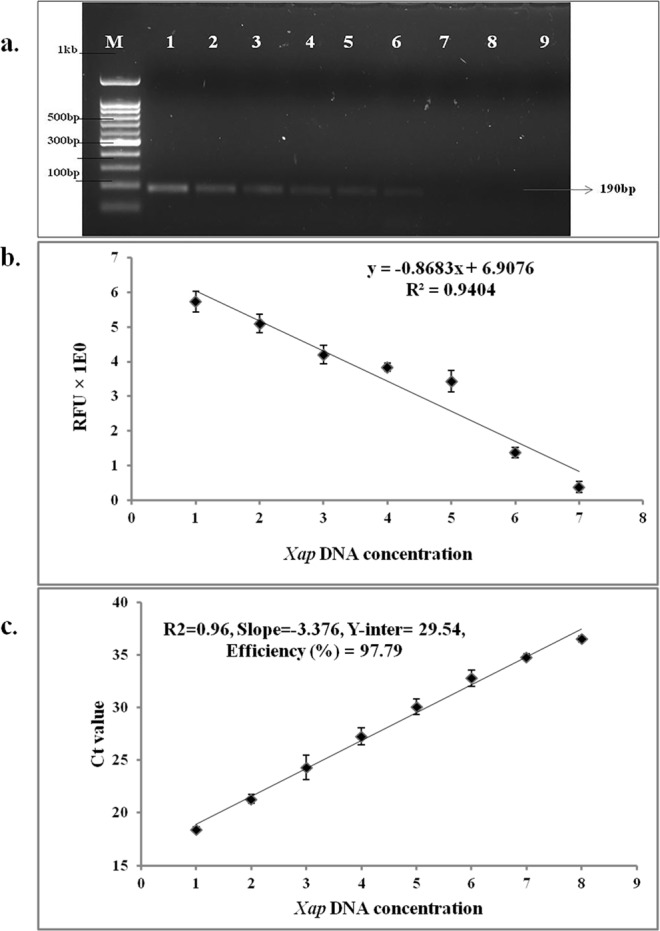
Sensitivity of *Xanthomonas axonopodis* pv. *punicae* detection in pomegranate by different molecular techniques. (**a**) PCR-AGE: Polymerase Chain Reaction coupled with Agarose Gel Electrophoresis. (**b**) PCR-CE: Polymerase Chain Reaction coupled with Capillary Electrophoresis. (**c**) qPCR: Real-time quantitative PCR, dpi: days post inoculation. (M-100 bp DNA ladder, 1–9 PCR reactions with 10 – fold serially diluted template DNA concentrations isolated from *Xap* cultures at 1–100 ng, 2–10 ng, 3–1 ng, 4–100 pg, 5–10 pg, 6-1 pg, 7–100 fg, 8–10 fg and 9-1 fg).

Different *Xanthomonas* effector genes (*XopQ*, *XopC*, *XopE*, *XopN*, *XopL*, *XopZ*, *XopL*) were evaluated as candidate targets for pathogen detection. *Xanthomonas* spp. specific primers were designed by checking primer pair specificity against genomes of pomegranate and other pathogens (*Ceratocystis fimbriata* and *Cercospora punicae)* that infect pomegranate. Primer pairs of *XopC*, *XopE*, *XopN*, *XopL*, *XopZ*, *XopL* either produced more than one amplicon or exhibited low PCR efficiency. *XopQ* primers amplified a 190 bp region in genomic DNA isolated from *Xap* and *Xap*-infected plants, but not in the healthy plant samples (Fig. 1) with PCR efficiency of 97.79% (Fig. 2c). *XopQ* primers amplified a single amplicon exhibiting a single peak in the dissociation melting curve in qPCR (Fig. S1). The *XopQ* primer pair amplified the same 190 bp in other isolates of *Xap* (Fig. S2) and other species of *Xanthomonas* such as *X*. *axonopodis* subsp. *citri*, and *X*. *campestris pv*. *campestris* that infect lemon and cabbage respectively. But, *XopQ* primer pair did not amplify in the pomegranate leaves infected with *Ceratocystis fimbriata* and *Cercospora punicae* that cause wilt and leaf spot in pomegranate (Fig. S3).

### Sensitivity of pathogen detection by PCR and qPCR

The sensitivity of different PCR diagnostic methods was tested using a 10-fold serial dilution of *Xap* genomic DNA ranging from 100 ng to 1 fg as a template for PCR. Amplification of *XopQ* gene using conventional PCR-AGE in 1.5% agarose gel reached a detection limit of 1 pg of template DNA (Fig. 2a). The detection sensitivity increased to 100 fg with the capillary electrophoresis system (PCR-CE) (Figs 2b, S4). The highest detection sensitivity was observed in qPCR with amplification of template concentrations as low as 10 fg (Fig. 2c).

### Early diagnosis of bacterial blight in pomegranate

Four methods of disease diagnosis were compared for early diagnosis of bacterial blight in pomegranate: visual symptoms-based disease severity analysis, PCR-AGE, PCR-CE and qPCR. For PCR-based assays, total genomic DNA was isolated from challenge and mock inoculated plant leaves at 2, 4, 6, 8, 12 and 16 days post inoculation (dpi), and 50 ng of template DNA was used in the PCR reaction.

**Figure 3:**
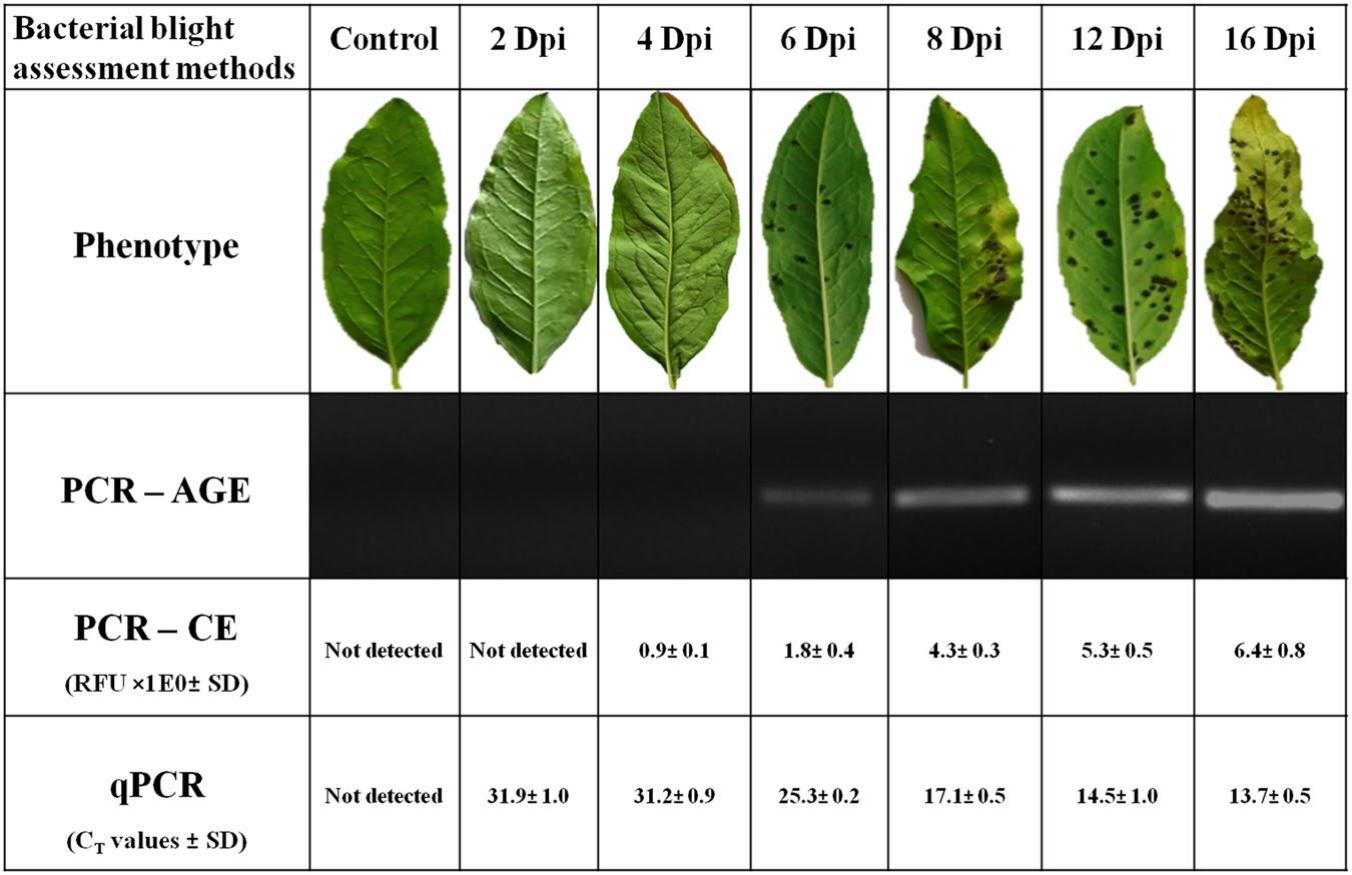
Detection of *Xanthomonas axonopodis* pv. *punicae (Xap)* in pomegranate at different days post inoculation by molecular diagnostic techniques. PCR-AGE: Polymerase Chain Reaction coupled with Agarose Gel Electrophoresis, PCR-CE: Polymerase Chain Reaction coupled with Capillary Electrophoresis, qPCR: Real-time quantitative PCR, dpi: days post inoculation, RFU: relative florescence unit.

In the conventional diagnosis based on the visual symptoms, the appearance of water-soaked lesions on the lower surface of spray-inoculated leaves were observed at 6 dpi for the highly susceptible variety ‘Bhagwa’. PCR-AGE detected *Xap* at 6 dpi similar to the symptom-based diagnosis. PCR-CE and qPCR detected *Xap* well before the appearance of symptoms at 4 dpi and 2 dpi respectively (Figs 3, S5, S6).

### Validation of early diagnosis of bacterial blight by qPCR

The ability of qPCR to detect the pathogen incidence at early stages of infection was validated using DNA extracted from non-symptomatic leaf samples collected from seven different orchards within a radius of 5 km (Table 1). Presence of *Xap* was detected by qPCR in five orchards, which later developed disease symptoms as water-soaked lesions on the lower surface of leaves at 6 to16 days after sample collection. *Xap* was not detected by qPCR in two orchards (Govindakoppa and Mallapur), that did not exhibit any bacterial blight symptoms until the last day of observation.

**Table 1:** Diagnosis of bacterial blight using qPCR in field samples collected from pomegranate orchards in Karnataka, India, lacking disease symptoms.

Sample	Location	GPS coordinates	C_T_ values ± SD	Symptom visibility (days after sample collection)
Orchard 1	Anagawadi	15°15′16.0″N, 75°37′15.8″E	26.27 ± 0.2	16
Orchard 2	Semikeri	16°11′36.8″N, 75°3414.9″E	11.16 ± 0.05	6
Orchard 3	Govindkoppa	16°12′06.1″N, 75°31′43.7″E	25.32 ± 0.12	13
Orchard 4	Herisamshi	16°11′53.9″N, 75°32′11.69″E	24.92 ± 0.073	13
Orchard 5	Govindkoppa	16°11′48.1″N, 75°32′17.5″E	Not detected	Not detected
Orchard 6	Tulsigeri	16°32′38.6″N, 75°33′51.5″E	24.41 ± 0.33	10
Orchard 7	Mallapur	16°18′55.6″N, 75°73′89.16″E	Not detected	Not detected
Challenge-inoculated plants (9 dpi)	University of Horticultural Sciences, campus field, Bagalkot	16°10′48.00″N, 75°42′0.00″E	24.89 ± 0.5	9
Water control	—		Not detected	

### Quantification and detection of *Xap* in pomegranate genotypes

**Figure 4:**
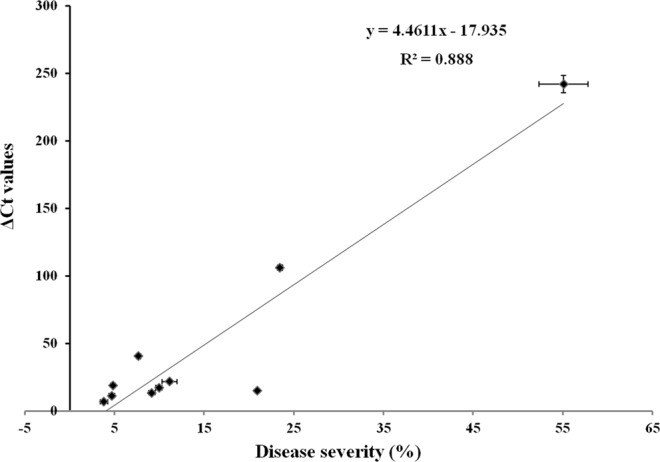
Comparison of pomegranate bacterial blight diagnosis by visual symptoms based disease severity and qPCR. Correlation between visual symptoms based disease severity (%) and qPCR (ΔCt values)

The disease severity based on the visual symptoms was compared with pathogen detection by quantification by ΔCt method using qPCR in bacterial blight tolerant pomegranate genotypes; IC318724, IC318735, IC318762, IC318734, IC318707, IC318706, ACC8, Nana, Daru and susceptible Bhagwa (Table 2). ΔCt values of *XopQ* correlated with the disease severity based on visual symptoms (R^2^ = 0.89, Fig. 4). qPCR was also able to differentiate pathogen load on the pomegranate genotypes that show slight differences in the symptomatic disease severity. Among the bacterial blight tolerant genotypes, IC318762, IC318735 and IC318724 did not show significant difference in the disease severity based on symptoms, whereas qPCR could significantly differentiate pathogen load in these three genotypes.

**Table 2:** Comparison between phenotype based disease severity and qPCR diagnosis of *Xanthomonas axonopodis* pv. *Punicae*.

Genotype	Disease severity (%)	ΔCt
IC318762	4.47^f^	11.39^fg^
IC318735	3.79^f^	6.8^g^
IC318724	4.75^ef^	19.09^de^
IC318734	9.16^cd^	13.54^ef^
IC318707	9.95^cd^	17.32d^ef^
IC318706	11.15^c^	21.73^d^
Acc8	7.63^de^	40.6^c^
Nana	23.49^b^	106.06^b^
Dharu	20.94^b^	15.0^ef^
Bhagwa	55.09^a^	241.9^a^
CD@0.05	2.93	6.6
CV	13.95	7.8
SEM	0.57	1.34

ΔCt: Values derived from qPCR analysis using DNA extracted from *Xap* infected leaves, CV: coefficient of variation, CD: critical difference, SEM: standard error of the mean. Means followed by same letter between groups in a column are not significant at a confidence interval of 5%.

## Discussion

Rapid and accurate detection of a pathogen at early and latent infection is essential for effective disease management. Disease identification by visual symptoms in the field is a simple and inexpensive approach. However, symptom-based diagnostics are subject to observer bias and errors and are unreliable at asymptomatic stage of infection. Most importantly, the symptoms of one disease can also be confused with those of other diseases^26^. Although culture-based morphological features of the pathogen are used for precise detection of the pathogen, these techniques depend on the “culturability” of the pathogen and are time consuming, laborious^27^. Molecular-based techniques such as PCR^23^, ELISA^28^ and qPCR^25^ have greatly contributed to the precise and early detection of pathogens during latent infection stages before any visible symptoms appear on plant parts.

**Figure 5:**
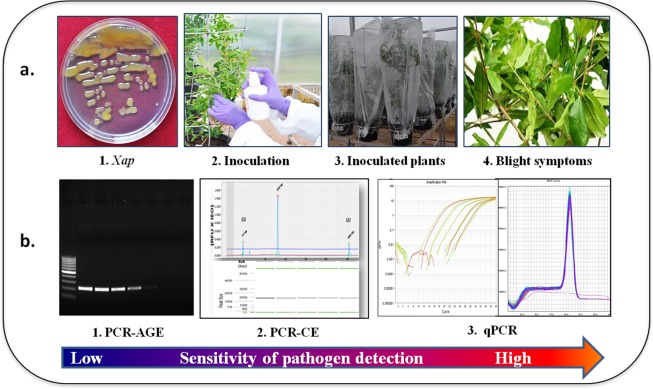
Overview of testing bacterial blight diagnosis in pomegranate by molecular techniques. (**a**) Pathogen challenge inoculation under greenhouse conditions. (**b**) Disease diagnosis by PCR, Capillary Electrophoresis, and qPCR.

Bacterial blight caused by *X*. *axonopodis* pv. *punicae* has become an increasingly serious threat for pomegranate cultivation causing yield losses up to 80% under epidemic conditions. Disease is diagnosed only when water-soaked lesions develop on the leaves 6 to 39 days after infection that develop into dark brown spots surrounded by bacterial ooze^6^. Bacteria on the leaf surface act as secondary inoculum that rapidly spreads through rain splash, irrigation water, pruning tools, humans and insect vectors^29^. Early diagnosis of the disease is helpful for disease management with low-dose pesticides^16^. Moreover, the excessive and continuous application of pesticides leads to the development of resistance by the pathogen. In this study, PCR based molecular diagnostic tools, PCR-AGE, PCR-CE and qPCR were evaluated for precise, rapid and early diagnosis of bacterial blight in pomegranate (Fig. 5).

*Xanthomonads* specific effector gene *XopQ* primers were used for detection of *Xap* in pomegranate by using PCR techniques. *XopQ* primers specifically amplified *Xap* in infected samples but did not amplify DNA from healthy or infected pomegranates with fungal diseases. *XopQ* primers amplified similar amplicon (190 bp) in other *Xanthomonas* species (Fig. S3). However, *X*. *axonopodis* subsp. *citri*, *X*. *campestris* pv *campestris* that are closely related to *Xap* failed to infect pomegranate (Figs S7 and S8). Thus, *XopQ* primers used in this study may be used for specific detection of *Xap* and diagnosis of bacterial blight in pomegranate. Usually ribosomal DNA (rDNA) of phytopathogens is targeted for detection and disease diagnosis. However, this type of DNA is evolutionarily conserved across genera and species and fails to discriminate well among closely related taxonomic species^30^. Bacterial genes coding for effectors are specific to pathogen species/races and precisely detect the targeted pathogen.

All three PCR methods (PCR-AGE, PCR-CE and qPCR) were useful in the detection of *Xap* in the bacterial blight infected samples. PCR-AGE is not suitable for early diagnosis of bacterial blight in the asymptomatic plants because of its low sensitivity, in addition to being labour intensive, time consuming and exposing users to hazardous carcinogenic chemicals like ethidium bromide^23^. PCR-CE is rapid as no manual preparation of gels and staining are required and is sensitive to detect low template DNA and is amenable to automation for quick screening of many samples^24^. Because of high resolution, PCR-CE is also suitable for simultaneous detection of multiple pathogens by multiplex-PCR^24^. Primers specific to other pathogens such as *Cercospora punicae* and *Ceratocystis fimbriata* that infect pomegranate could be used in combination with *XopQ* primers in multiplex PCR and analysed on PCR-CE for simultaneous diagnosis of multiple diseases.

Real-time quantitative PCR (qPCR) was sensitive to detect *Xap* infection in pomegranate within 2 dpi in the challenge-inoculated samples and was able to detect the pathogen at latent stage in the asymptomatic field samples (Fig. 3 and Table 1). The versatile qPCR may be used for diagnosis of bacterial blight latent infection in the asymptomatic fields for regular disease survey and in the nurseries for producing healthy plant material and certification of the plant material^25^. Relative pathogen quantification by qPCR could be used as an alternative to disease severity analysis by visual symptoms as the ΔCt values were highly correlated with the symptoms-based disease severity (Fig. 4). The current method of measurement of bacterial blight disease severity in pomegranate is based on counting the infected leaves out of total number of leaves and grading based on visual observation of bacterial spots on each leaf^6^. One-year pomegranate plant that is commonly used for disease severity analysis bears 600 to 1000 leaves. Hence disease severity analysis based on visual symptoms is laborious and time consuming. Moreover, disease grading of leaves is biased and error prone by the researcher. Accurate and reliable pathogen biomass quantification methods are also necessary for studying plant pathogen interactions *in vivo*, to determine plant resistance towards a pathogen or to estimate the aggressiveness of a particular pathogenic strain^31,32^. There are certain germplasm and genetic mapping population which differ slightly in their response to pathogen infection. Sensitive qPCR differentiates these germplasms and it enhances the efficiency of selection, which is otherwise not possible through conventional disease severity analysis.

Molecular pathogen detection methods developed in the present study may be used in commercial disease diagnostic laboratories and research institutes for rapid and early diagnosis of bacterial blight in commercial farms for effective disease management. PCR-CE and qPCR are reliable for early detection of bacterial blight under field conditions, accurate quantification of disease severity in breeding lines, and disease forecasting and disease indexing in nurseries as preventive measures.

## Methods

### Plant production

Pomegranate genotypes Bhagwa, IC318724, IC318735, IC318762, IC318734, IC318707, IC318706, ACC8, Nana and Daru with variable levels of bacterial blight resistance were used in this study. ‘Bhagwa’ is a widely cultivated variety in southern India and is highly susceptible to bacterial blight. Other genotypes were developed at National Research Centre on Pomegranate, Solapur, India and reported to exhibit bacterial blight tolerance. Five plants of each variety were grown in a greenhouse maintained at 30 ± 2 °C and 60–70% relative humidity. One year old plants were used for artificial challenge inoculation. The experiment was conducted as a randomized complete design with three replications. One-month old cabbage and one-year old grafted lemon were procured from the Horticulture nursery, Main Horticulture Research and Extension Centre, University of Horticultural Sciences, Bagalkot, India.

### Isolation and purification of *Xap*

A pure colony of *Xanthomonas axonopodis* pv. *punicae* (*Xap*) was isolated from bacterial blight infected fruits from the pomegranate orchards, maintained at University of Horticultural Sciences, Bagalkot, India. Leaves infected with bacterial blight were surface sterilized with 0.1% HgCl_2_, and washed twice with sterile water and approximately 2 mm diameter lesions were cut and macerated in sterile water. Bacteria were cultured on nutrient glucose agar (NGA) medium and incubated at 27 °C ± 2 °C for 3–4 days^6^. Single colonies were sub-cultured on NGA medium and incubated at 27 °C ± 2 °C; pinheaded, yellowish colonies, which appeared after 48 h of incubation, were selected for inoculum preparation and streaked on NGA medium.

### Total gDNA isolation from different samples

Genomic DNA from *Xap* and *Xap spp*. was isolated from a single colony-inoculated culture grown for 72 h at 28 °C in NG broth^6^ and total DNA from diseased (Bacterial blight, *Cercospora* leaf spot, wilt) and healthy leaf samples of pomegranate was isolated^23^ and quantified using a NanoDrop 1000 spectrophotometer (ND-1000, ThermoFisher, MA, USA).

### PCR amplification of *Xap* genes

For PCR-based diagnosis of bacterial blight in pomegranate, six *Xap* effector genes were selected: *XopQ* (JN993529), *XopC* (KT277099), *XopE* (KT351085), *XopN* (JQ034360), *XopL* (CP009025), and *XopZ* (KT277100). Primers were designed for these genes using primer-BLAST^33^. Primer specificity for *Xap* was checked against the genome assembly of *Punica granatum* (ASM220158v1), *Cercospora* (all nucleotides of taxid: 29002) and *Ceratocystis fimbriata* (custom in house assembly of PRJNA381691). Primers of the six effector genes that amplified only in *Xap*, but not in pomegranate, *Cercospora* and *C*. *fimbriata*, were selected for further analysis. *XopQ* primers (Forward primer- 5′-GCGAGGAACTTGGAATGCTC-3′ and reverse primer 5′-AGGTCGAAGGCTTTTTGCG-3′) that exhibited best PCR efficiency with product size of 190 bp was used for bacterial blight diagnosis in pomegranate.

Amplification of *XopQ* primers in different isolates of *Xap and* different species of *Xanthomonas* was evaluated by isolating genomic DNA from their respective pure cultures. Species specificity was tested on total genomic DNA of *Xanthomonas campestris* pv. *campestris*, and *Xanthomonas citri* subsp. c*itri*.

For amplification of a target gene, PCR was set up in 15 µl of reaction mix containing 50 ng of DNA, 1X PCR buffer, 200 μM dNTPs, 0.5 µMoles each of forward and reverse primer and 0.3 U of taq DNA polymerase (New England Biolabs, Ipswich, MA, USA). *Xap* specific effector genes were amplified by initial denaturation at 94 °C for 4 min, 35 cycles of denaturation at 94 °C for 15 s, annealing at 58 °C for 30 s, extension at 72 °C for 45 s, followed by final extension at 72 °C for 10 min in a thermocycler (Eppendorf AG, Hamburg, Germany). For PCR-AGE, 5 µl of amplified PCR products was resolved on 1.5% agarose gel using a submerged horizontal electrophoresis system (Bio-Rad, Hercules, California, USA). The products were stained with ethidium bromide, and gel images were observed and photographed using a Gel Logic 212 Pro imaging system (Gel Logic 212 PRO, Carestream, USA). For PCR-CE, 15 µl of amplified PCR product (injection volume 3 µl) was analysed using a QIAxcel genetic analyser (Qiagen, Hilden, Germany), using the OM800 method with preassembled QIAxcel DNA high-resolution cartridge, QX alignment Marker 15 bp–600 bp and QX size marker 25 bp–600 bp.

### Quantitative real-time PCR (qPCR)

qPCR was evaluated for rapid diagnosis of bacterial blight infection using DNA extracted from blight-infected pomegranate leaves, with *Xap* DNA as the positive control. qPCR was performed in a 10 µl reaction mixture comprising 1X SYBR Green PCR Master Mix (Applied Biosystems, Foster City, CA), 0.5 µM each of forward and reverse primer and 50 ng of DNA isolated by a modified CTAB method used as the template. qPCR was performed in a Step OnePlus Real-Time PCR System (Applied Biosystems, Foster City, California, USA) using *XopQ* as the target gene and *GAPDH* (KF856731, forward primer 5′-AGCCTACAACCAAACATCAAGC-3′ and reverse primer 5′-GGTGCCGAGTTCATTGTGGA-3′ as the reference gene to normalize sample-to-sample variation. The following PCR conditions were used: initial denaturation at 95 °C for 2 min, followed by 40 cycles of denaturation at 95 °C for 15 s and annealing at 60 °C for 1 min. Following PCR amplification, melt-curve analysis of the amplicons was conducted from 60 °C to 95 °C, and data were collected at 0.3 °C intervals.

PCR efficiency of *XopQ* primers was tested by plotting the standard curve created by means of plotting the logarithm of known initial concentrations of *Xap* DNA over six orders of tenfold dilution (100 ng to 1 pg) versus cycle threshold (C_T_) values. The standard curve was developed by plotting the C_T_ values against known serial dilutions of quantified DNA from *Xap*. Amplification efficiency was calculated using the formula E = (10(−1/slope)^−1^) × 100, where E is the amplification efficiency and the slope is the log of template concentrations versus C_T_.

### Testing sensitivity of bacterial blight diagnosis by PCR and qPCR

The sensitivity of *Xap* detection by PCR-AGE, PCR-CE and qPCR was evaluated with tenfold serially diluted *Xap* DNA ranging from 100 ng to 1 fg as template in their respective 10 µl PCR reaction mixture. *Xap* specific primer *XopQ* was used to amplify the genomic DNA of *Xap*. PCR reaction mix and PCR conditions were similar to those previously described for PCR and qPCR.

### Challenge inoculation of pomegranate with *Xap* and disease severity analysis

Bacterial inoculum for challenge inoculation was prepared by scraping bacterial colonies from the culture plates using a sterile loop followed by suspension in sterile distilled water. Final concentration of bacterial suspension was adjusted to 0.4 OD at 600 nm (4 × 10^8^ CFU/ml) for inoculation.

One-year-old healthy pomegranate plants were spray-inoculated with water as mock inoculation and the freshly prepared *Xap* suspension using an airbrush (Model Badger-200.3, Deluxe set™; Badger Air Brush Co., Franklin Park, IL, USA). To facilitate infection, inoculated plants were covered with moist transparent plastic bags to maintain high moisture followed by removal at 24 h post inoculation (hpi). The experiment was conducted in three replicates for each genotype with five plants in each replicate.

### Cross inoculation studies of *Xanthomonas spp* on Pomegranate

The pure bacterial strain of *Xanthomonas campestris* pv. *Campestris* was obtained from Indian Type Culture Collection (ITCC), Indian Agricultural Research Institute, New Delhi India. The pure bacterial strain of *Xanthomonas citri* subsp. *citri* was obtained from the Department of Biotechnology, University of Mysore, Karnataka India. Both the bacterial strains were sub-cultured on NGA medium for mass production. Bacterial inocula preparation and challenge inoculation were carried out similar to *Xap* inoculation study described above. In addition to inoculation on pomegranate plants (Bhagwa variety), *Xanthomonas campestris* pv. *campestris* and *Xanthomonas citri* subsp. *citri* were also inoculated to their respective host plants cabbage (*Brassica oleracea* var. *capitata*) and Citurs (*Citrus lemon*). Appearance of typical symptoms on the leaves was monitored up to 20 days post-inoculation.

#### Disease diagnosis by visual symptoms

Bacterial blight in different pomegranate genotypes with varied levels of genetic resistance was diagnosed by scoring appearance of water-soaked lesions on the leaf surface at 2, 4, 6, 8, 12, and 16 dpi. Disease was scored on 100 leaves on each genotype. Water-soaked lesions were graded on a 1–5 scale (with 1 smaller and 5 larger lesions), and percent disease severity was calculated at 16 dpi when symptoms were visible on all the genotypes tested^34^.

$${\rm{Percent}}\,{\rm{disease}}\,{\rm{severity}}=\frac{{\rm{Number}}\,{\rm{of}}\,{\rm{infected}}\,{\rm{leaves}}\times {\rm{Grade}}\,\mathrm{obtained}\,}{{\rm{Total}}\,{\rm{number}}\,{\rm{of}}\,{\rm{leaves}}\times \mathrm{Maximumgrade}\,}\times 100$$

### Early detection of bacterial blight in pomegranate

Bacterial blight susceptible variety ‘Bhagwa’ was challenge-inoculated with *Xap*. Leaf samples were collected from the inoculated plants at 2 day intervals from 2 to 16 dpi. Total genomic DNA was extracted from the leaf samples, and 50 ng of DNA was used as template for disease diagnosis using PCR and qPCR. In parallel, observations of appearance of blight symptoms were recorded and disease severity was calculated. The experiment was conducted on three separate plants of ‘Bhagwa’ as biological replicates.

### Relative quantification of *Xap* in pomegranate genotypes

Rapid bacterial blight diagnosis methods were validated in different genotypes of pomegranate with varied levels of bacterial blight resistance. Ten genotypes with three replicates (Table 2) along with the susceptible ‘Bhagwa’ were spray-inoculated with *Xap* as described previously. Disease in inoculated plants was diagnosed by visual symptoms (percent disease severity), PCR methods (PCR-AGE and PCR-CE) and qPCR at 16 dpi. Sensitivity and rapidness in detecting the disease were compared.

### Bacterial blight diagnosis in pomegranate orchards

Leaf samples from seven pomegranate orchards around Bagalkot, Karnataka, India (Table 1), which did not show any visual blight disease symptoms, were collected for validation of the method for early detection of bacterial blight under field conditions. Leaf samples from 5 trees from each orchard were randomly collected and pooled for DNA extraction. Selected orchards were more than two years old and were pruned in Nov-Dec 2017 (Ambe bahar treatment) to induce new flush and flowers. Total DNA was extracted from the leaf samples and analysed using qPCR. Observations of appearance of blight symptoms were recorded at three-day intervals until symptoms appeared in the field conditions.

## Supplementary information

Supplementary files for the manuscript

## References

[1] Johanningsmeier SD, Harris GK (2011). Pomegranate as a functional food and nutraceutical source. Annu. Rev. Food. Sci. T..

[2] Anonymous, Horticultural statistics at a glance, National Horticulture Board, Govt. of India, **46** (2017).

[3] Pal R, Babu KD, Singh N, Maity A, Gaikwad N (2014). Pomegranate Research in India–Status and future challenges. Progr. Hort..

[4] Hingorani M, Mehta P (1952). Bacterial leaf spot of pomegranate. Indian Phytopathol.

[5] Ramesh C, Ram K (1991). Studies on bacterial blight (*Xanthomonas campestris* pv. *punicae*) of pomegranate. Indian Phytopatholo..

[6] Sharma J (2017). Pomegranate bacterial blight: symptomatology and rapid inoculation technique for *Xanthomonas axonopodis* pv. *punicae*. J. Plant. Pathol..

[7] Akhtar M, Bhatti MR (1992). Occurrence of bacterial leaf spot of pomegranate in Pakistan. Pak. J.Agric. Res.

[8] Petersen Y, Mansvelt E, Venter E, Langenhoven W (2010). Detection of *Xanthomonas axonopodis* pv. *punicae* causing bacterial blight on pomegranate in South Africa. Australas. Plant. Path..

[9] Icoz S (2014). First Report of Bacterial Blight of Pomegranate Caused by *Xanthomonas axonopodis* pv. punicae in Turkey. Plant Dis.

[10] Sharma, K., Sharma, J. & Jadhav, V. In *Recent Advances in Diagonosis and Management of plant Disease* 119–126 (Springer 2015).

[11] Yuan Z (2018). The pomegranate (*Punica granatum* L.) genome provides insights into fruit quality and ovule developmental biology. Plant Biotechnol. J..

[12] Kumar R, Mondal KK (2013). XopN-T3SS effector modulates in plant growth of *Xanthomonas axonopodis* pv. *punicae* and cell-wall-associated immune response to induce bacterial blight in pomegranate. Physiol Mol Plant P.

[13] Soni M, Mondal KK (2018). *Xanthomonas axonopodis* pv. *punicae* uses XopL effector to suppress pomegranate immunity. Journal of integrative plant biology..

[14] Sharma, K. & Sharma, J. Diseases of pomegranate and their management. *Plant Pathology in India*, **74** (2011).

[15] Jadhav, V. T. & Sharma, K. K. Intergated management of diseases in pomegranate. Souvenir and abstracts 2nd international symposium on pomegranate and minor including Mediterranean fruits, UAS Dharwad 23–27 (2009).

[16] Benagi, V. L., RaviKumar, M. R & Nargund V. B. Threat of bacterial blight on pomegranate in India – Mitigation by an integrated approach. II International Symposium on the Pomegranate 113–116 (2012).

[17] Lalithya KA, Manjunatha G, Raju B, Kulkarn IMS, Lokesh V (2017). Plant growth regulators and signal molecules enhance resistance against bacterial blight disease of pomegranate. Journal Phytopathoogyl..

[18] Mondal KK, Mani C (2012). Investigation of the antibacterial properties of nanocopper against *Xanthomonas axonopodis* pv. *punicae*, the incitant of pomegranate bacterial blight. Ann. Microbiol.

[19] Ambadkar CC, Dhawan AS, Shinde VN (2015). Integrated management of bacterial blight disease (oily spot) of pomegranate caused by *Xanthomonas axonopodis* pv. Punicae. Int. J. Plant Sci.

[20] Chavan NP (2016). Biocontrol potential of actinomycetes against *Xanthomonas axonopodis* pv. *punicae*, a causative agent for oily spot disease of pomegranate. Biocontrol Sci. Techn.

[21] Gargade VA, Kadam DG (2015). *In vitro* evaluation of antibacterial potential of *Pongamia pinnata* L. against *Xanthomonas axonopodis* phytopathovar of bacterial blight of pomegranate (*Punica granatum*). Int. J. Curr. Microbiol. Appl. Sci.

[22] Martinelli F (2015). Advanced methods of plant disease detection. A review. Agron. Sustain. Dev.

[23] Mondal KK (2012). The reliable and rapid polymerase chain reaction (PCR) diagnosis for *Xanthomonas axonopodis* pv. *punicae* in pomegranate. Afr. J. Microbiol. Res..

[24] Wu Xu-long, Xiao Lu, Lin Hua, Yang Miao, Chen Shi-jie, An Wei, Wang Yin, Yao Xue-ping, Yang Ze-xiao, Tang Zi-zhong (2017). A Novel Capillary Electrophoresis-Based High-Throughput Multiplex Polymerase Chain Reaction System for the Simultaneous Detection of Nine Pathogens in Swine. BioMed Research International.

[25] Garces F, Gutierrez A, Hoy J (2014). Detection and quantification of *Xanthomonas albilineans* by qPCR and potential characterization of sugarcane resistance to leaf scald. Plant Dis.

[26] Mahlein A-K, Oerke E-C, Steiner U, Dehne H-W (2012). Recent advances in sensing plant diseases for precision crop protection. Eur. J. Plant. Pathol.

[27] Lievens B, Thomma BP (2005). Recent developments in pathogen detection arrays: implications for fungal plant pathogens and use in practice. Phytopathology.

[28] Wang Z (2012). The development and application of a Dot-ELISA assay for diagnosis of southern rice black-streaked dwarf disease in the field. Viruses.

[29] Benagi, V. I. Bacterial Blight of Pomegranate, https://www.apsnet.org/publications/imageresources/Pages/FI00143.aspx.

[30] McCartney HA, Foster SJ, Fraaije BA, Ward E (2003). Molecular diagnostics for fungal plant pathogens. Pest Manage. Sci..

[31] Yogendra, K. N. *et al*. Transcription factor StWRKY1 regulates phenylpropanoid metabolites conferring late blight resistance in potato. *J Exp Bot*, erv **434** (2015). 10.1093/jxb/erv434 PMC4765800 26417019

[32] Annegret R, Imre ES, DNA-based A (2016). real-time PCR assay for robust growth quantification of the bacterial pathogen *Pseudomonas syringae* on *Arabidopsis thaliana*. Plant methods.

[33] Ye J (2012). Primer-BLAST: a tool to design target-specific primers for polymerase chain reaction. BMC bioinformatics.

[34] Singh NV (2015). Genetic diversity and association mapping of bacterial blight and other horticulturally important traits with microsatellite markers in pomegranate from India. Mol. Genet. Genomics.

