# Supplementary material for: Reliable and early diagnosis of bacterial blight in pomegranate caused by Xanthomonas axonopodis pv. punicae using sensitive PCR techniques
Source: Sci Rep. 2019 Jul 12;9:10097. doi: 10.1038/s41598-019-46588-9 (PMC6625978; doi:10.1038/s41598-019-46588-9)
Supplement: Supplementary file 1 — Supplementary files for the manuscript [file 41598_2019_46588_MOESM1_ESM.pdf]

Reliable and Early diagnosis of bacterial blight in pomegranate caused by *Xanthomonas axonopodis* pv. *punicae* using sensitive PCR techniques

Pushpa Doddaraju[a], Pavan Kumar[a], Raghavendra Gunnaiah[b], Abhishek A. Gowda[a], Veeresh Lokesh[a] , Parvati Pujer[b] , and Girigowda Manjunatha[a*]

[a]Bio-control Lab, Directorate of Research, [b]Dept. of Biotechnology and Crop Improvement, University of Horticultural Sciences, Bagalkot, Karnataka, India

Corresponding Author[*]: Girigowda Manjunatha (gmanjunath2007@gmail.com)

Supplementary files for the manuscript

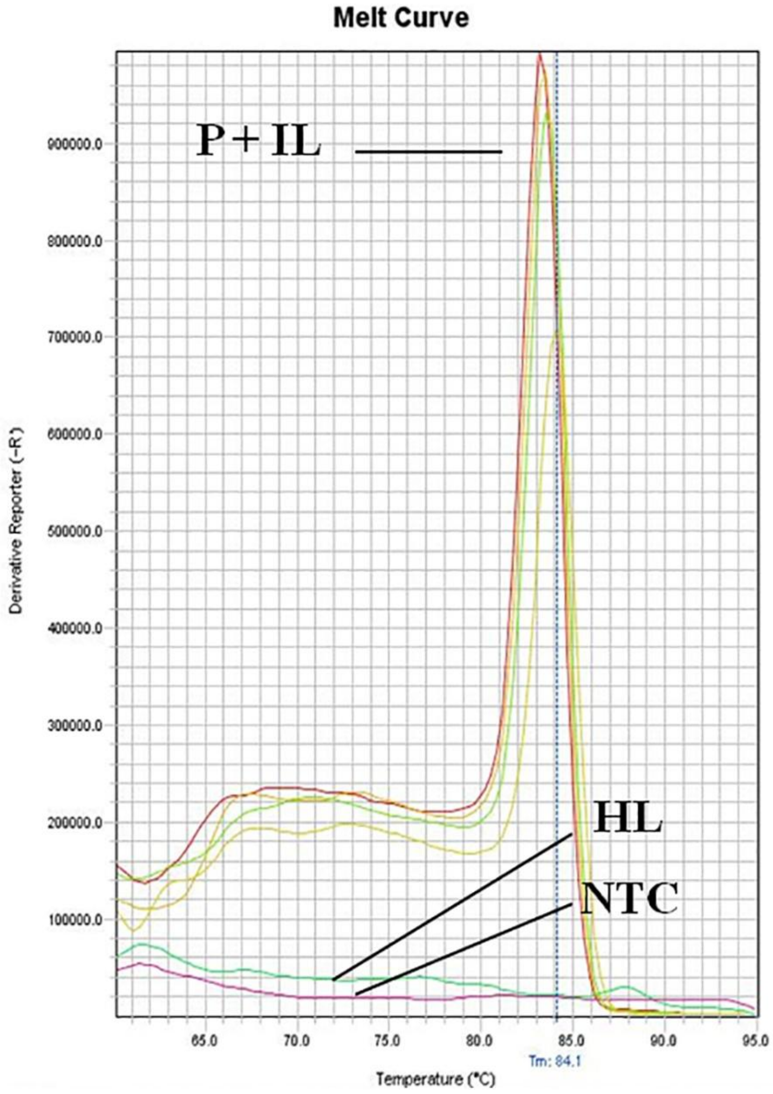

Figure S1. Dissociation melt curve analysis of *XopQ* primers in qPCR used for detecting *Xanthomonas axonopodis* pv. *punicae* (*Xap*). DNA extracted from pure culture of *Xap* (P), infected leaf (IL), healthy leaf (HL), NTC- no template control.

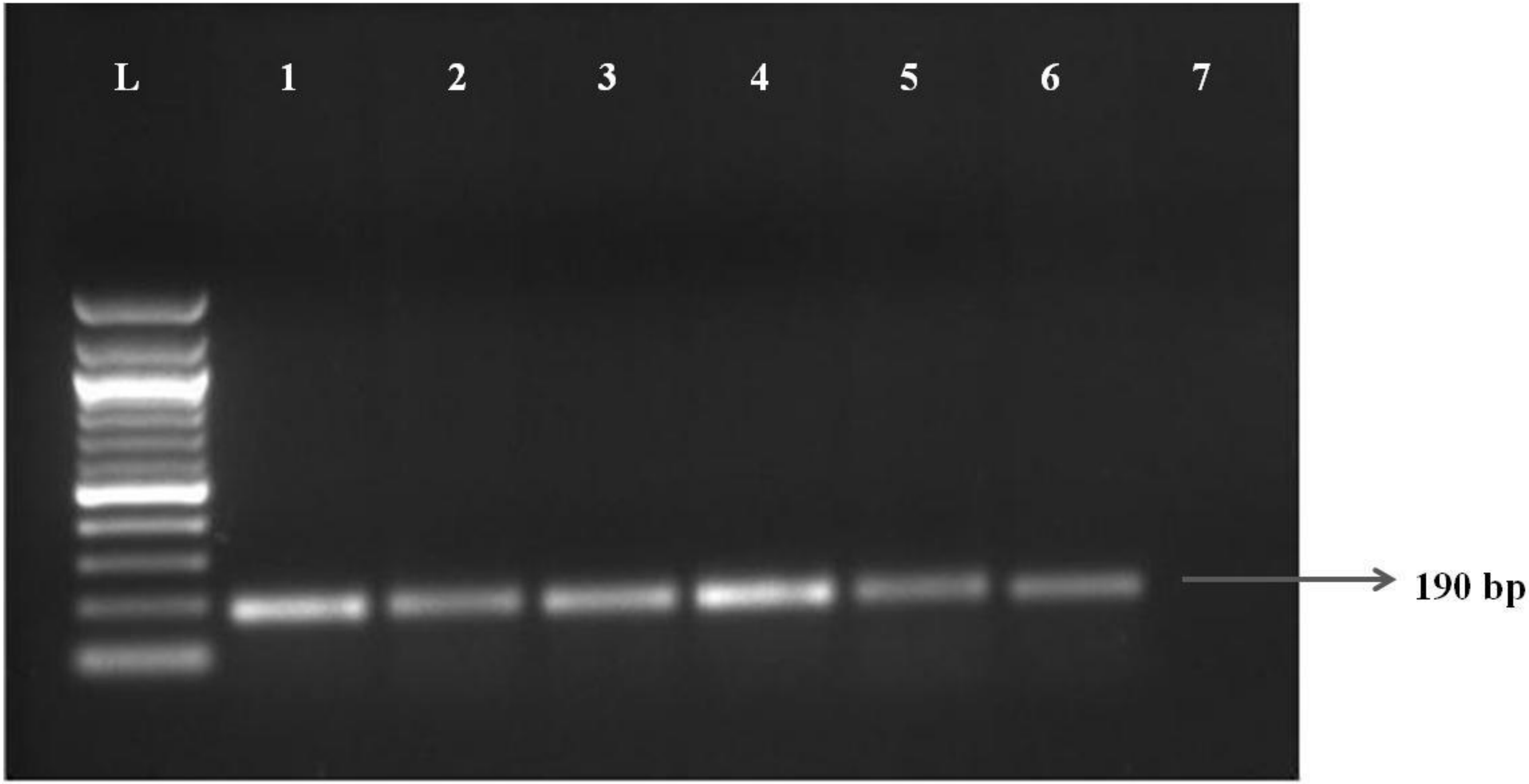

Figure S2. PCR amplification of *XopQ* primers (190 bp) using DNA extracted from different isolates of *Xanthomonas axonopodis* pv. *punicae* collected from different geographical regions of India . L- 100 bp DNA ladder, 1- Isolate 1 (Bagalkot, Karnataka), 2- Isolate 2 (Kaladagi, Karnataka), 3- Isolate 3 (Navanagar, Karnataka), 4- Isolate 4 (Vijaypura, Karnataka), Isolate 5 (Bhuj, Gujarat), 6- Isolate 6 (Solapur, Maharashatra), 7- no template control.

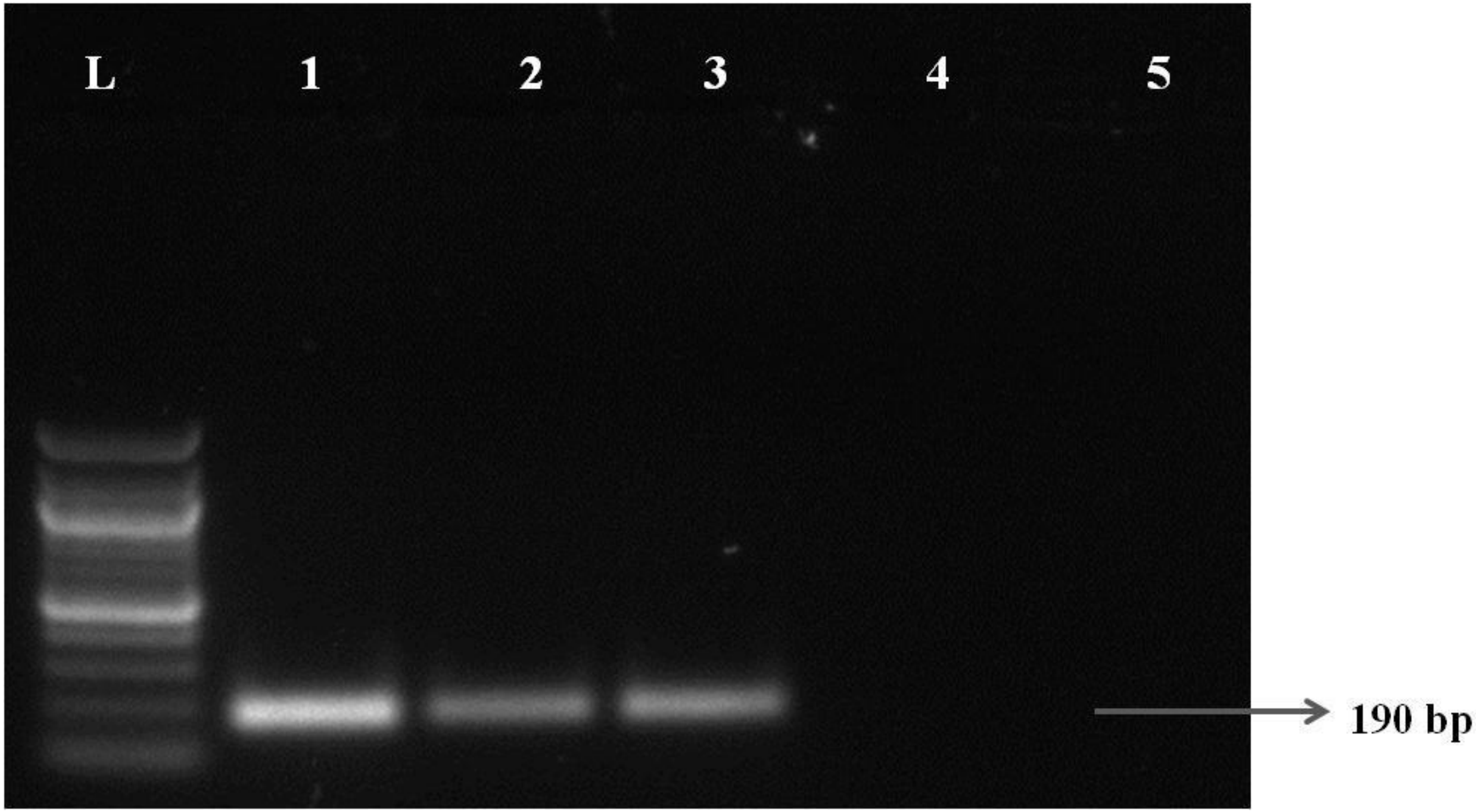

Figure S3. PCR amplification of *XopQ* primers (190 bp) using DNA extracted from plant leaves infected with different species of *Xanthomonas*. L- 100 bp DNA ladder: 1- *Punica granatum* L. infected with *Xanthomonas axonopodis* pv. *punicae*, 2- *Brassica oleracea* infected with *Xanthomonas campestris* pv. *campestris*, 3- *Citrus limon* infected with *Xanthomonas axonopodis* subsp. *citri* , 4- *Punica granatum* L. infected with *Cercospora punicae*, 5- *Punica granatum* L. infected with *Ceratocystis fimbriata*.

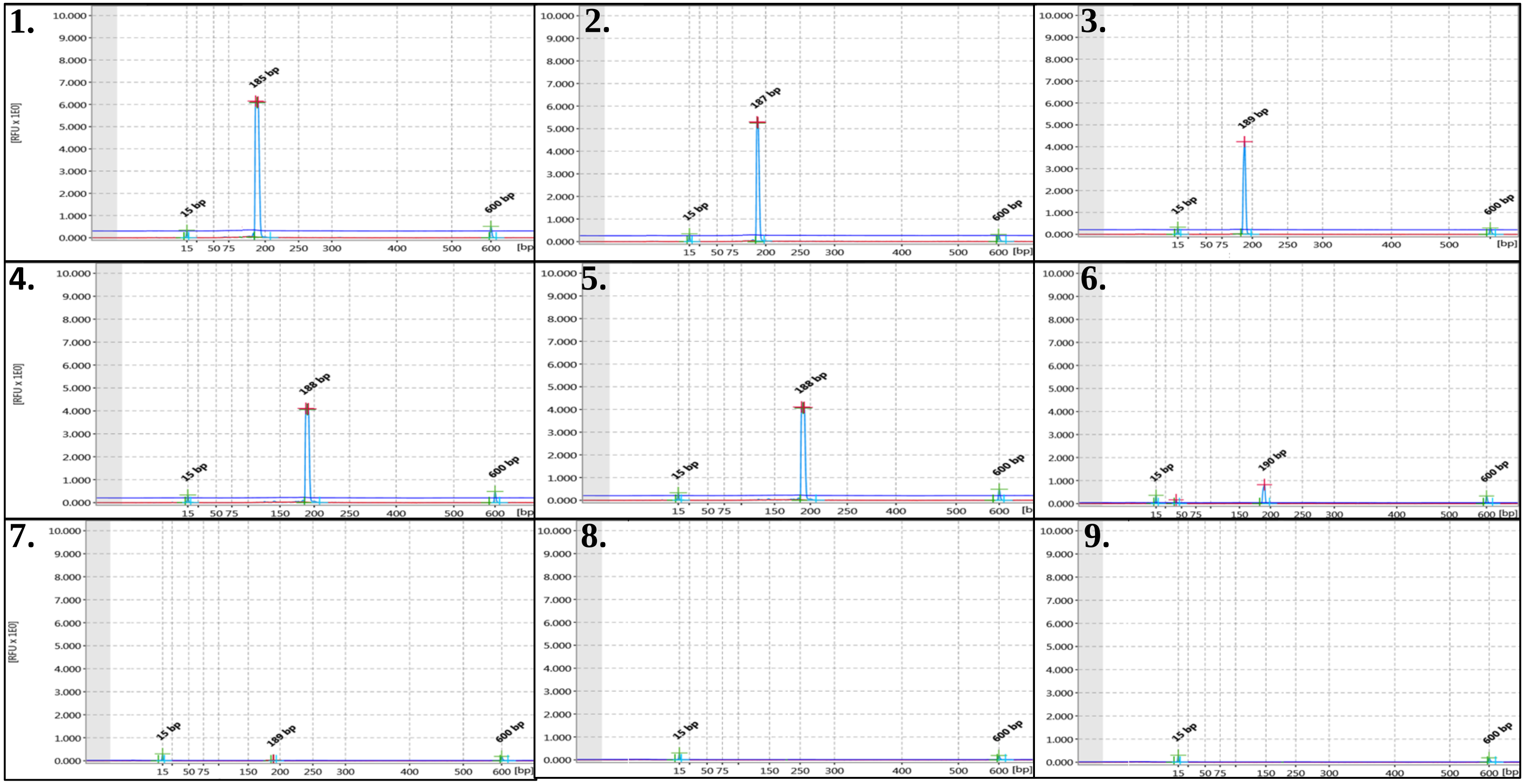

Figure S4. Capillary electrophoresis (QIAxcel), electropherograms of PCR product amplified using *XopQ* primers with different concentration of *Xap* DNA (1-100ng, 2-10ng, 3-1ng, 4-100pg, 5-10pg, 6-1pg, 7-100fg, 8-10fg, and 9-1fg).

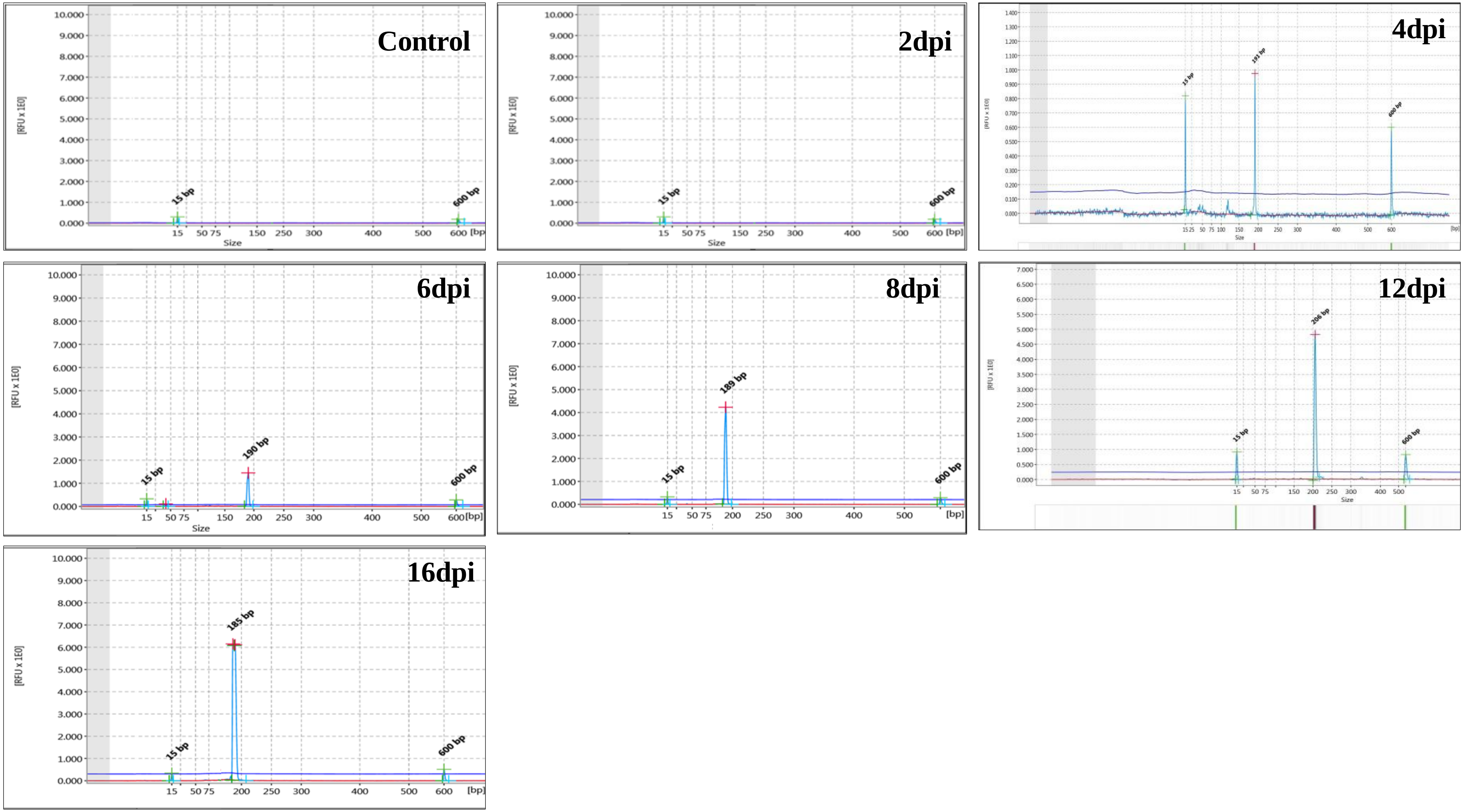

Figure S5. Detection of bacterial blight in pomegranate at different days of post inoculation (dpi) of *Xanthomonas axonopodis* pv. *punicae* by PCR-CE:Polymerase chain reaction coupled with capillary electrophoresis.

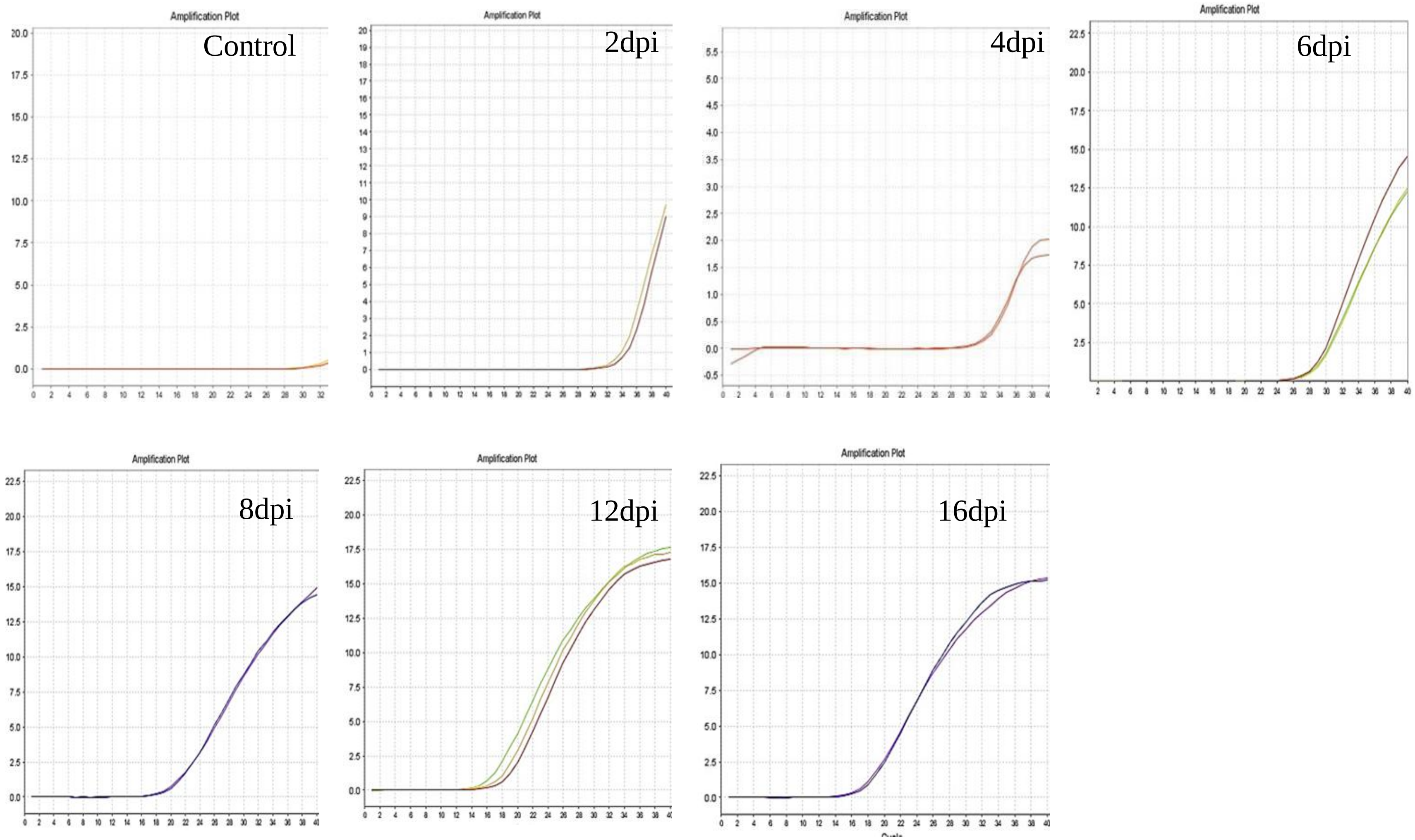

Figure S6. Detection of bacterial blight in pomegranate at different days of post inoculation (dpi) of *Xanthomonas axonopodis* pv. *punicae* by qPCR: Real time quantitative PCR.

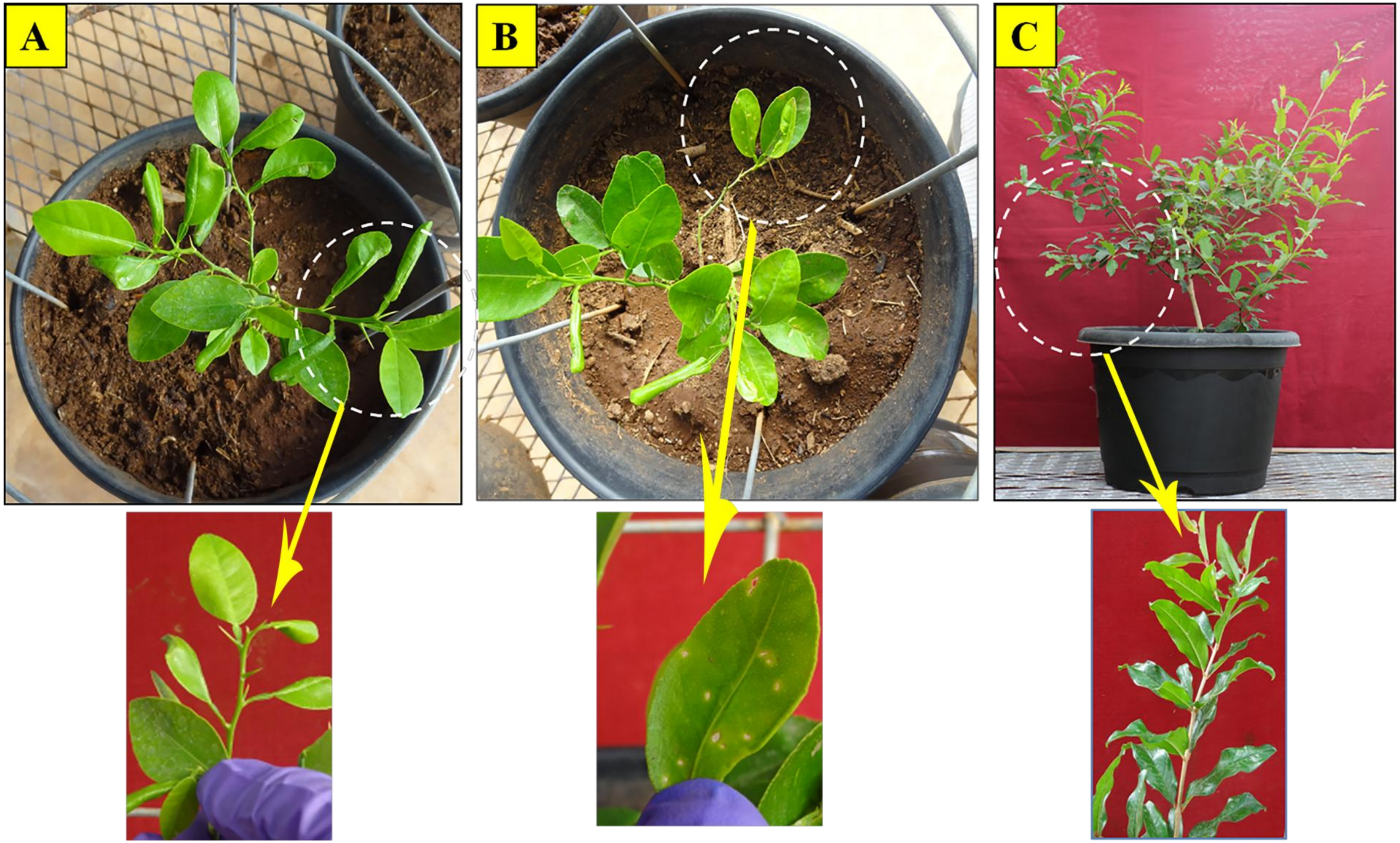

Figure S7. **Pathogenecity test of *Xanthomonas citri* subsp. *citri* on lemon (*Citrus limon)* and pomegranate.** A. Lemon inoculated with water (control) B. Lemon inoculated with *Xanthomonas citri* subsp. *citri*., C. Pomegranate inoculated *with Xanthomonas citri* subsp. *citri*.

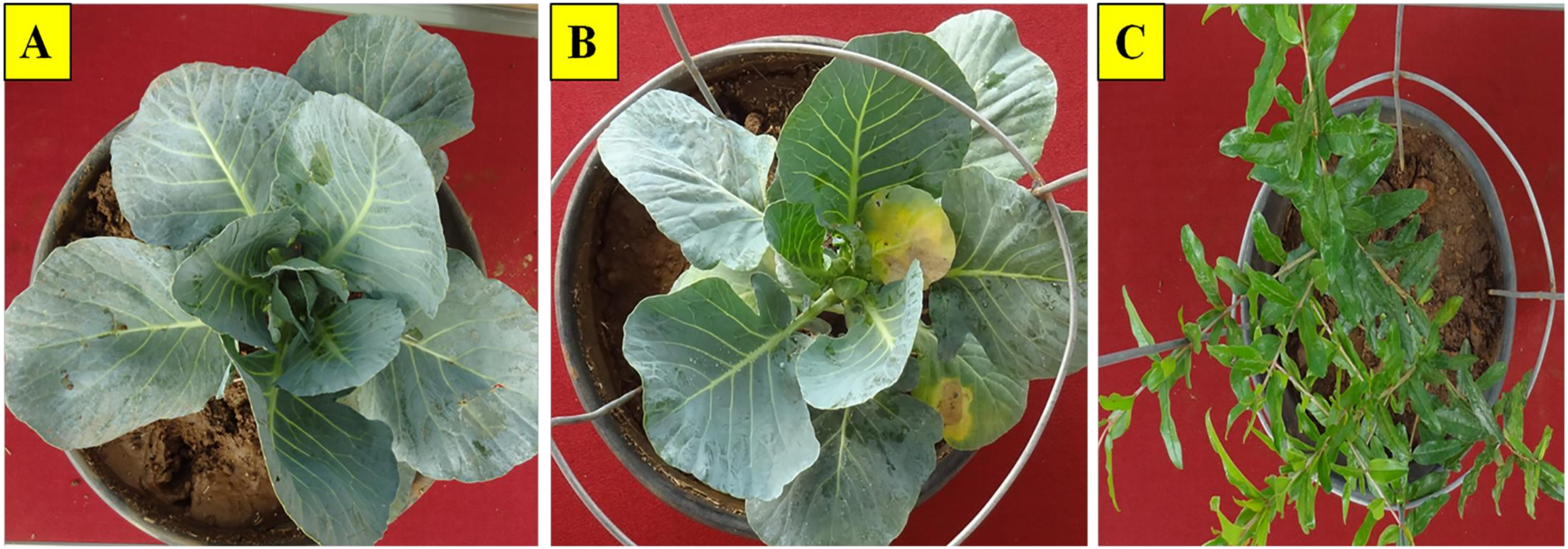

Figure S8. Pathogenecity test of *Xanthomonas campestris* pv. *campestris* on cabbage and pomegranate. A. Cabbage inoculated with water (control), B. Cabbage inoculated with *Xanthomonas campestris* pv. *campestris*. C. Pomegranate inoculated with *Xanthomonas campestris* pv. *campestris.*
